# Analysis of Plastic Improvement and Interference Behavior in Current-Assisted Riveting of CFRP Laminates

**DOI:** 10.3390/ma15051673

**Published:** 2022-02-23

**Authors:** Zhenchao Qi, Ziqin Zhang, Yexin Xiao, Xingxing Wang

**Affiliations:** 1 Department of Mechanical and Electrical Engineering, Nanjing University of Aeronautics and Astronautics, Nanjing 210016, China; zhangzqin@nuaa.edu.cn (Z.Z.); wangxx@squ.edu.cn (X.W.); 2 AECC Hunan Aviation Powerplant Research Institute, Zhuzhou 412002, China; 1137974075@nuaa.edu.cn

**Keywords:** rivet, current density, interference CFRP, plasticity

## Abstract

In order to improve the joint performance of a titanium alloy rivet connecting aircraft CFRP structure and promote the wide application of ordinary titanium alloy rivets in the aviation field, the ductility of a Ti45Nb rivet was improved using a current-assisted method in this paper. Through experiments, the mechanical behavior and temperature during the riveting process were monitored, and the variation rules of interference and damage were studied in detail. The results show that a current within 16.5 A/mm^2^ can effectively reduce the riveting pressure requirement, and the maximum engineering stress is reduced by nearly 22%. As the current density increases, the softening effect is obvious, but as the processing time increases, the softening effect has an upper threshold. The current-assisted method can significantly increase the interference fit level, and the uniformity of riveting can be improved by nearly 30%. The outlet burr height of a joint obtained by new technology meets the relevant standards. When the current density is too large or the action time is long, the damage pattern and mechanism at different depths of hole have obvious regional differences.

## 1. Introduction

Carbon fiber reinforced polymer (CFRP) has been widely used in the aerospace and aviation fields due to its high specific strength and specific stiffness, and its structural connection technology has also attracted much attention [1]. Titanium alloy fasteners are often used to replace aluminum alloy fasteners in aircraft composite structural connections to reduce galvanic corrosion between dissimilar materials in the structure [2,3]. Due to the large forming force of titanium alloy rivets, there are problems, such as easy cracking of the upsetting head and uneven distribution of nail rod interference, which limit the wide application of titanium alloy rivets in aeronautical engineering [4]. Therefore, exploring a method that can effectively improve the uniformity of the interference distribution and reduce the riveting forming force is the key to promoting the widespread application of ordinary titanium rivets in the aviation field.

The electrical treatment of metal is a feasible way to increase the workability of metal. While improving the plasticity of metal, it also has the advantages of fast temperature rise [5], rapid processing time [6], and multi-effect coupling [7]. In the field of current-assisted metal forming, many scholars have studied the deformation ability and forming energy requirements of metal materials during the forming process. Perkins et al. [8] had performed electric-assisted compression experiments on aluminum, copper, stainless steel, and titanium alloys, respectively, and found that various metals exhibited high deformation ability after being subjected to electrical treatment. Ross et al. [9] also found that applying DC current to the compression and tensile deformation process of Ti-6Al-4V can greatly reduce the force required for the deformation and set up isothermal experiments to verify the electroplastic effect in electric processing. Salandro et al. [10] had studied the current-assisted bending experiments of stainless steel, and the results show that the current-assisted bending can significantly reduce the load required for bending.

The above studies have verified that, through the coupling of electrical and thermal effects, electrical treatment could conspicuously improve the plasticity of titanium alloys and decrease the energy requirements for forming. Considering that CFRP composite materials have a shorter heat resistance compared to metal materials, the current-assisted method is an effective energy application means for CFRP laminated structure riveting. When it is introduced into the riveting process of CFRP composite materials, the multiple effects of current assist can be used to solve the problems of excessive riveting force of the titanium alloy rivets and uneven interference distribution in the temperature resistance range of CFRP [11].

It is worth noting that the riveting process under the condition of self-resistance heating of titanium rivets is a dynamic process of rapid [12], multi-field action [13] and multi-interface restriction [14]. When current is applied to the riveting process of a titanium alloy rivet, the plasticity change will directly affect the flow performance and filling level of the metal structure during riveting deformation, which will further affect the interface combination of the assembly.

In view of the above problems, the current-assisted riveting test was performed on a self-designed current-assisted riveting device. Titanium rivet riveting tests, with different current densities and action durations, were set up to analyze the deformation behavior of titanium rivets under different electrical parameters. The changes in rivet plasticity and riveting interference of titanium alloy rivets in the current-assisted riveting process are studied.

## 2. Materials and Methods

### 2.1. Materials

**Figure 1:**
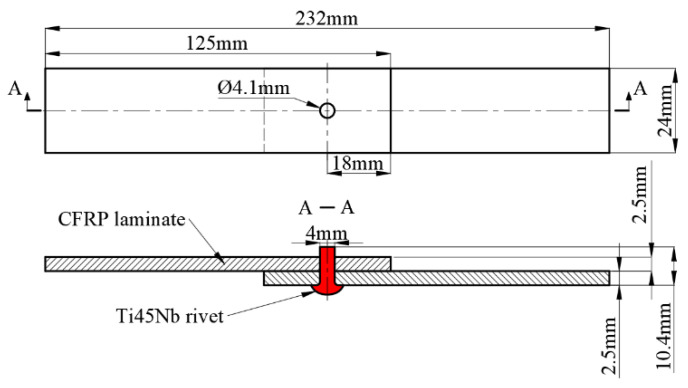
Single lap riveted joint.

The research material in this study is Ti45Nb-based titanium alloy rivet. Riveting experiment was performed on the unidirectional carbon fiber reinforced composite lapped plate. The lapped plate is made of continuous fibers in a quasi-isotropic layup. The lapped plate refers to the standard in ASTM D5961 as a single-shear lap joint, the specific dimensions are shown in Figure 1 [15]. Firstly, the relative position of the laminated specimen was fixed, and phenolic resin backing plate was added at the outlet side of the borehole to improve the quality of the borehole. Then, we used a dagger drill to make a hole with a spindle speed of 2000 rpm and a feed rate of 0.03 mm/r. The diameter of the hole is slightly larger than the rivet’s shank. The exact parameters are shown in Table 1.

### 2.2. Experiment Methods

**Figure 2:**
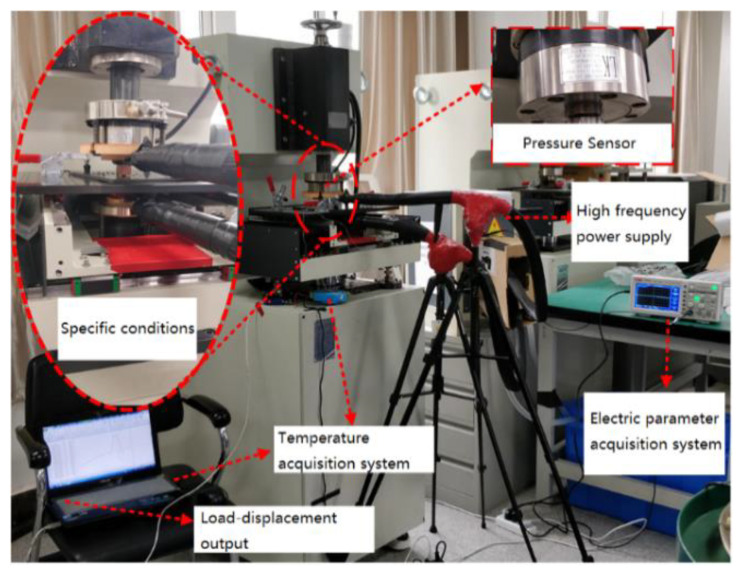
The experimental platform for current-assisted riveting.

Figure 2 shows the experimental equipment for current-assisted riveting experiment. The high-frequency power supply (DXK-12V2000A, Shanghai Runfeng Company, Shangai, China) is used to generate high-frequency current (33,000 Hz) of the pulse signal to electrically treat the titanium alloy rivets during riveting. For safety reasons, an insulation device is designed. The temperature acquisition module (K-type thermocouple) is responsible for monitoring the temperature during the riveting process. The pressure and displacement information during the riveting process can be collected by the riveting machine and can be out as log file.

The riveting device is used for riveting the titanium alloy rivets with current assistance, and high-speed acquisition channel of the riveting device control system (TRIO) can collect the displacement information generated by the servo motor. Combined with the pressure data generated by the pressure sensor (LKH-117/5T, Nanjing Lanke Automation Equipment Co., Ltd., Nanjing, China), the deformation behavior of the titanium alloy rivets riveting process can be collected in real time.

**Figure 3:**
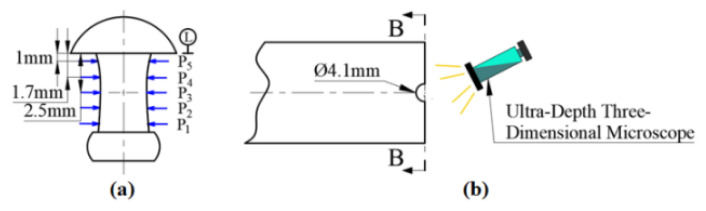
Location of interferometric measurement and damage observation. (**a**) Schematic diagram of interference measurement position. (**b**) Schematic diagram of damage observation.

In order to evaluate the quality of the connection area of the riveted joint, the riveted composite material laminate was cut with a diamond wheel cutting machine (T7-800, Zhengzhou Golden Dragon Machinery Factory, Zhengzhou, China), the titanium alloy rivet was taken out, and the interference level of the rivet was measured with a digital vernier caliper. The measuring position is shown in Figure 3. The Ultra-Depth Three-Dimensional Microscope (HiROX RH-2000, Koshi Corporation of Japan, Ashiya, Japan) was used to observe the microscopic appearance of the CFRP material in the connection area to analyze the thermal and mechanical damage of the joint.

### 2.3. Scheme

Two groups of single-factor current-assisted riveting schemes with different current density and different processing time are designed in this study. The current density is defined as the ratio of the current to the initial area of the rivet screw rod section, and the processing time is defined as the duration of the current-treated rivet. The specific parameters are shown in Table 2. The current density is divided into 6 levels from 0 A/mm^2^ to 18 A/mm^2^; the electrical time is divided into 5 levels from 0 s to 60 s. The temperature of the current-assisted riveting process is collected using a temperature acquisition card. Using pulse power generators to control different levels of parameters, current-assisted riveting experiments were performed on titanium alloy rivets. The riveting parameters are attached after Table 2. The riveting keeps the same pressure riveting parameters, the maximum pressure is 14,200 N, and the pressure riveting speed is 0.2 mm/s. The experiment was repeated three times for each group.

## 3. Results and Discussions

### 3.1. Plasticity of Ti45Nb Rivets

**Figure 4:**
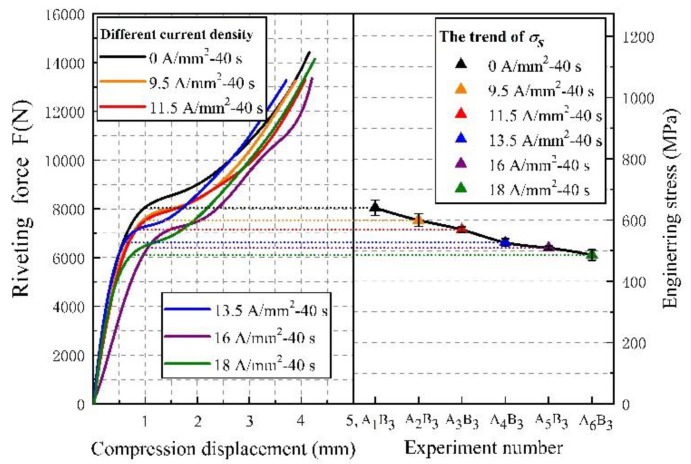
Titanium rivets riveting force–displacement curve at different current densities.

First, we processed the log data output from the riveting machine. The force–displacement curves of the riveting process are shown in Figure 4. In this paper, the yield strength is calculated numerically at the inflection point of the curve. The stress is obtained by the ratio of the riveting force to the initial nail section area of the rivet. All the curves show that the compression behavior of Ti45Nb titanium alloy rivets has typical compression characteristics of plastic materials. After current action of the rivets, the deformation ability of rivet materials during riveting is further enhanced.

With the increase in current density, the yield limit of the titanium rivet material is significantly reduced, and the flow stress of riveting is significantly lower than that of no current-assisted riveting. Taking the yield point as the evaluation index, plasticity can be quantitatively assessed, the yield stress σ_s_ changes under different electrical parameters are shown in Table 3. After the current treatment of the rivet for 40 s with 18 A/mm^2^, the plasticity improvement of the titanium rivets reached maximum. The corresponding engineering stress decreased by 147 MPa and the stress reduction accounted for 22.9% of the yield stress σ_s_ without current assist. It can be predicted that as the current density increases, the plasticity of titanium alloy rivets will still improve, but the Joule heat caused by electricity may have a negative effect on the CFRP pore wall. The plasticity of the metal was greatly improved after current assist. This means that the rivet can enter the plastic deformation earlier when the rivet is pressed, and the flow and filling of the material is more sufficient.

**Figure 5:**
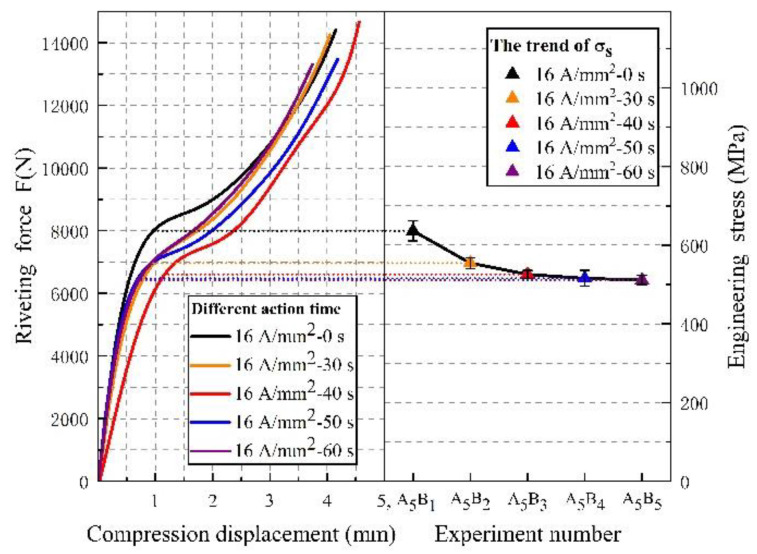
Titanium rivets riveting force–displacement curve at different electric action times.

We changed the current duration from 30 s to 60 s and the force–displacement curves of the riveting process are shown in Figure 5. It is not difficult to find that the yield limit of material under current pretreatment can be reduced to a certain extent, and the plasticity is enhanced by electric excitation, which is similar to the previously obtained results. The difference is that, with the extension of the electric treatment time, the deformation characteristic in the compression process is that the softening degree of Ti45Nb rivet material gradually presents a threshold value, and the yield strength is very close after 50 s. The corresponding yield stress σ_s_ at each electrical action time is shown in Table 4. After the current pretreatment of the rivet for 40 s with 16 A/mm^2^, the corresponding engineering stress decreased by 123 MPa compared to the yield stress σ_s_ without current. Respectively, the stress reduction during electrical treatment for 50 s and 60 s is 125 MPa and 129 MPa, which means that as the electrical processing time increases, the degree of plasticity improves, in a process from strong to weak.

**Figure 6:**
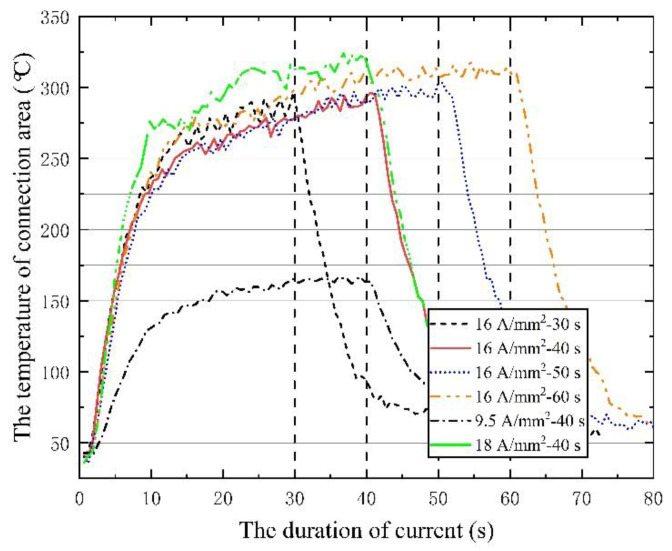
Temperature rise monitoring curve.

In order to analyze the differences of the above plasticity phenomena, the temperature rise curves of the center connection area under different action electrical parameters were compared and analyzed. The temperature response at the rivet bar, measured by K-type thermocouple, is shown in Figure 6. All the temperature rise curves have a characteristic: the self-resistance heating temperature of the rivet will have a peak value. When the duration of the current reaches 30~40 s, the heat generation and heat dissipation will reach a balance, and the temperature will be maintained at a specific level. Therefore, the maximum temperature under different electric durations is basically the same, the internal heat storage of the rivet is close, and the thermal processing characteristics shown are also similar. The main difference under different current densities is the level of saturation temperature. The temperature of high current density is higher, and the corresponding thermal softening during riveting is also more significant.

### 3.2. Riveting Interference Behavior

For a riveting structure, the level of interference caused by riveting and its uniformity largely determine the service capability of connection [16,17]. When the interference fit level of riveting is small, the ability of the structure to bear the applied load decreases. When the interference fit level of riveting is too large or the distribution in the hole is not uniform, it will over-extrude the hole wall and cause in-plane buckling or interlayer splitting of the carbon fiber, which will also affect the service ability of the connection structure [18,19,20]. Finally, the current-assisted riveting joint was cut open, and the deformation of the rivet after compression was measured, according to the position shown in Figure 3. Through Formulas (1) and (2), the absolute interference fit level Δ and the average level $\bar{\Delta }$ of interference were calculated, respectively, and the results are shown in Table 5.

(1) $$\Delta =d^{\prime }-D$$

(2) $$\bar{\Delta }=\frac{1}{n}\sum_{i=1}^{n}\left(d^{\prime }-D\right)$$

where *n* is the total number of interference measurement points; *d*’ is the diameter of the rivet after compression; *D* is the aperture of the connecting plate before riveting.

The range coefficient *P* and variation coefficient *CV* were introduced to quantitatively evaluate the uniformity of rivet interference distribution in the hole. The values of *P* and *CV* were obtained from Equations (3) and (4), respectively.

(3) $$P=(\Delta_{\max}-\Delta_{\min})/\bar{\Delta }\times 100\%$$

(4) $$CV\%=\frac{1}{\bar{\Delta }}\sqrt{\frac{1}{n}\sum_{i=1}^{n}{\left(\Delta_{i}-\bar{\Delta }\right)}^{2}}\times 100\%$$

where Δ_max_ and Δ_min_ are the maximum and minimum values of the detected interference fit level on the rivet rod; Δ_*i*_ is the absolute interference fit level at the *i*-th measurement point on the rivet rod; $\bar{\Delta }$ is the average level of interference on the rivet rod.

**Figure 7:**
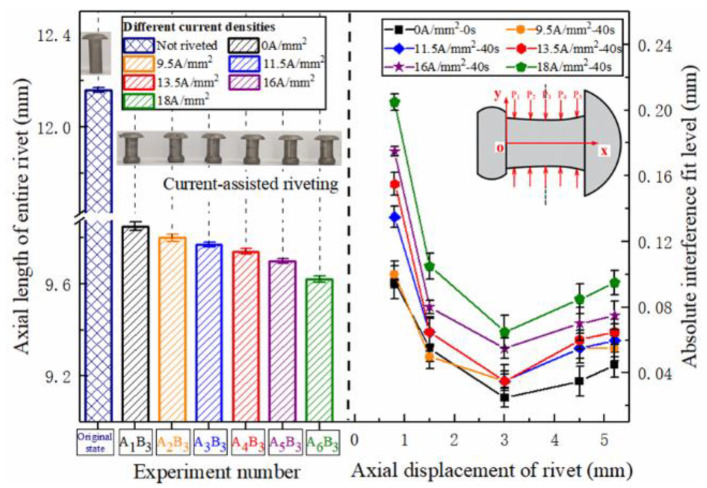
Rivet length and axial interference distribution under different current densities.

The variation of the entire rivet length and interference distribution after riveting under different current densities is shown in Figure 7. It is not difficult to find that, as the current density increases, the softening effect of current on the metal gradually increases, and the level of rivet interference has increased. The metal of the rivet outstretched near the hole wall has a stronger flow ability, and more of the metal structure flows into the hole, resulting in a substantial increase in interference in the area near the driving head of the rivet. The area near the rivet cap is not the main deformation area, and the interference fit level increases slightly. When the current density exceeds 9.5 A/mm^2^, the level of interference fit at P_1_ begins to increase sharply.

**Figure 8:**
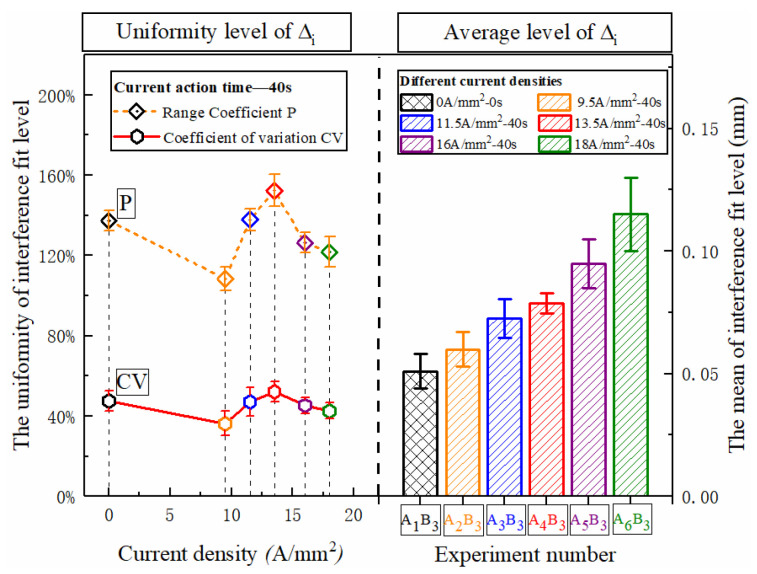
Uniform distribution of rivet interference under different current densities.

**Figure 9:**
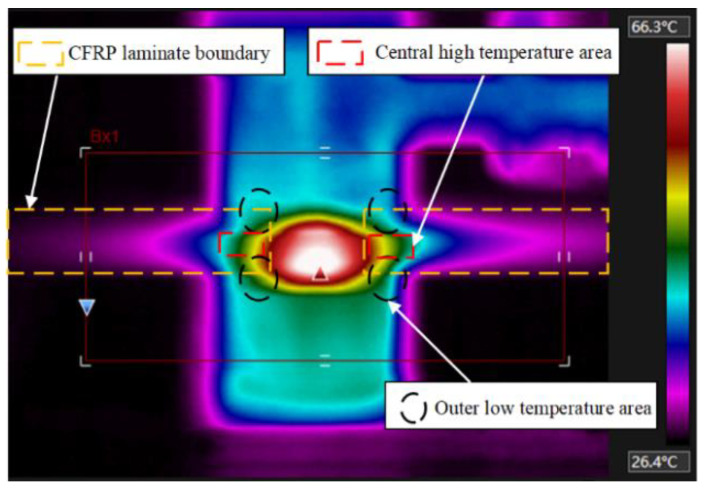
Thermal imaging during current processing.

The uniformity of the interference fit level under different current densities is shown in Figure 8. As the current density increases, the deformability of the metal increases, and the overall interference fit level increases approximately linearly. The dispersion degree of the axial interference distribution in the rivet fluctuates. The interference uniformity at 9.5 A/mm^2^ is relatively best. The reason for the fluctuation is largely due to the characteristics of the composite matrix material. When the current density is low, the connection area is at a relatively low temperature, about 150 °C, the CFRP has not been softened. In order to further understand the temperature field distribution law of current-assisted riveting, the current riveting experiment was performed according to the process parameters mentioned above, and the thermal imager (FLIR A310, Shanghai Pumeng Optoelectronic Technology Co., Ltd., Shanghai, China) was used for auxiliary observation. The thermal imager in the current processing process shows that under the action of Joule heating (Figure 9), the center temperature is higher than the two sides of the hole inlet and outlet, and the temperature gradient is tens of degrees. This difference has run through the entire current processing process.

As the temperature increases, the temperature difference between the central area and the two sides will further increase. The main reason for the difference in axial temperature distribution is that the electrodes (die and punch) contacting at both ends of the rivet have better thermal conductivity, while the weak heat dissipation capacity of the center of CFRP can easily lead to the accumulation of heat in the center. The accurate temperature difference cannot be accurately measured by thermal imaging cameras or thermocouples due to the infrared loss and space limitations. Xiao et al. [21] pointed out in previous related studies that the temperature *T*(*t*,*z*) in the hole is a distributed parabola to both ends, with the center of the *z* axis as vertex, which satisfies Equation (5). The central Joule heat influence factor *f_t_*(*t*) reaches a fixed value of central saturation temperature under a given electrical circuit and heat exchange environment, as shown in Equation (6).

(5) $$T(t,z)=f_{t}(t)\left[1-a(J)z^{2}\right]+T_{R}$$

(6) $${\left.f_{t}(t)\right|}_{t\to \infty }=\frac{fD\rho_{s}SJ^{2}{\left(1-\bar{\varepsilon }+0.25{\bar{\varepsilon }}^{2}\right)}^{2}}{2\pi K_{f}\ln(d_{1}/d)}$$

where, *z* is the distance in direction of the central axis of the rivet rod and zero is located in the center of rod, *a*(*J*) is the axial temperature transfer gradient factor, and the magnitude of the gradient factor is related to the current density, which satisfies the formula $a=1.05\times 10^{-3}J^{2}$, *T_R_* is the room temperature, *S* and *ρ*_*s*_ are the sectional area and resistivity of the rivets, respectively, *f*, *D* and *J* are the frequency, single-pulse time and current density of the pulse current, respectively, *K_f_* is the thermal conductivity of the composite, $\bar{\varepsilon }$ is the axial strain value, *d*_1_/*d* is the radial heat transfer coefficient, which represents the heat transfer range in the material surface and is related to the current density. The fitting form is shown in (7).

(7) $$d_{1}/d(J^{2})=4.77785+0.03219J^{2}+0.00104J^{4}-3.90627\times 10^{-6}J^{6}$$

**Figure 10:**
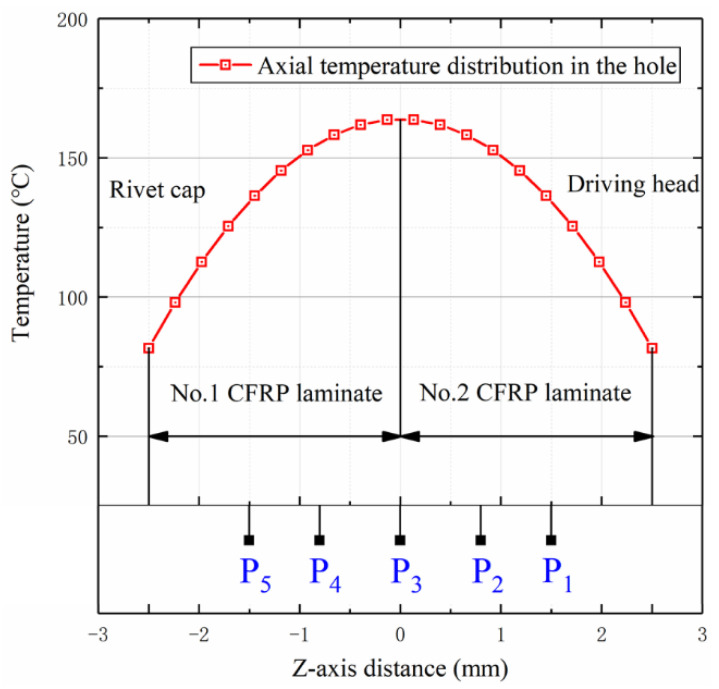
Temperature distribution at different depths in the hole.

Therefore, for the current treatment condition of 9.5 A/mm^2^, the relevant parameters in Table 6 were used to calculate the axial distribution of the rivet temperature. The temperature distribution from −2.5 mm to 2.5 mm on the rivet rod is shown in Figure 10. According to the predicted results of the model, the temperature at the inlet and outlet decreases by about 70 °C, relative to the central high temperature area, which leads to significant differences in the stiffness of CFRP materials in different areas [22,23]. This temperature difference is more accurate than the monitoring data of the infrared thermal imaging device. The high temperature in the center promotes the radial expansion capacity of the metal, while the lower temperature on both sides of the inlet and outlet still has a high confinement interference ability, which leads to the overall uniformity.

**Figure 11:**
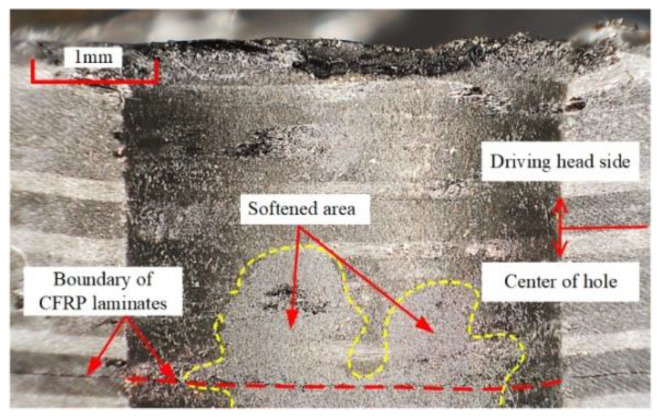
The Hole center softening diagram at 16 A/mm^2^.

It is important to note that when the current density exceeds 9.5 A/mm^2^, the uniformity of interference begins to decrease (Figure 8). The reason for this difference was not the change in temperature gradient of the inlet and outlet, but because the temperature at P_3_ in the connection area began to exceed 200 °C; the temperature at the inlet and outlet also began to rise above 130 °C. The increase in overall temperature greatly weakens the confinement ability of the CFRP hole wall [24,25]. Therefore, at the exit of the second CFRP plate, near the driving head side, the large deformation capacity of metal is difficult to fully restrict, and a high level of interference at point P_1_ is formed. At this point, the mechanical behavior at the P_1_ point is much more sensitive to high temperature than other areas in the hole. With the further increase in the current density, the temperature of the hole center increases further, and the center of the CFRP material also begins to soften (Figure 11), resulting in an increase in interference in the center of the CFRP hole, and the overall uniformity will drop slightly.

**Figure 12:**
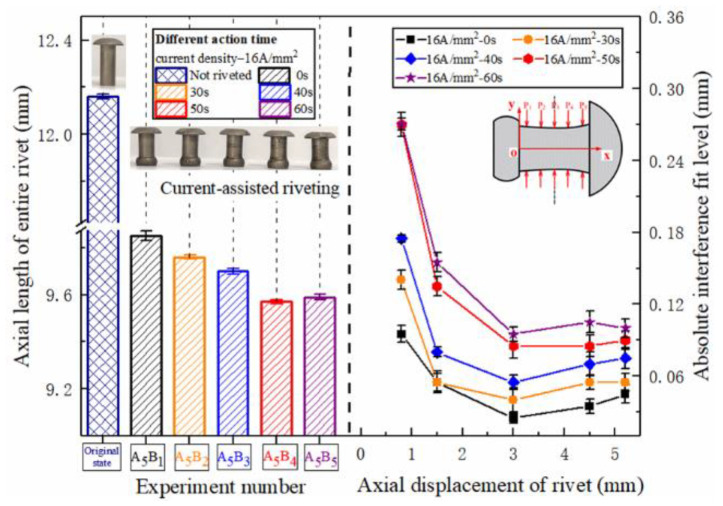
Rivet length and axial interference distribution under different action times.

**Figure 13:**
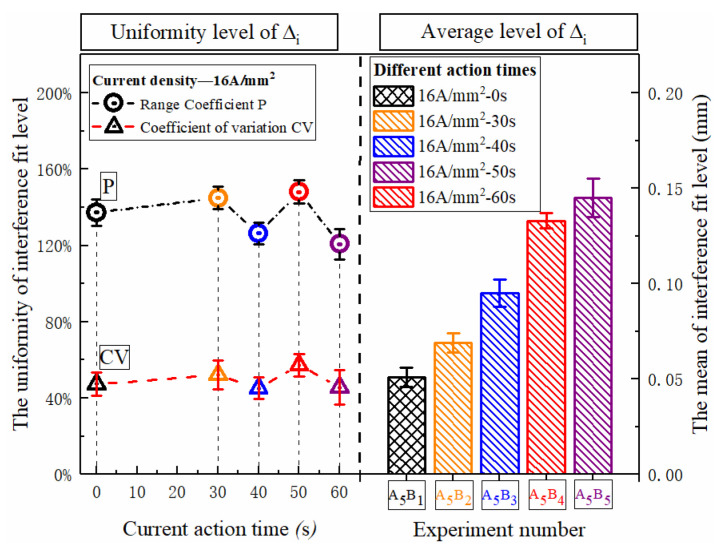
Uniform distribution of rivet interference under different action times.

The distribution of the entire rivet length and interference distribution under different action times is shown in Figure 12. With the extension of current treatment time, the amount of interference at various parts of the rivet rod also increased continuously. When the action time exceeded 40 s, the amplitude increased sharply. The interference distribution of 50 s and 60 s is basically the same, because the temperature after 40 s has essentially been saturated, as shown in Figure 6, and the temperature in the connection area reaches the highest. The phenomenon in Figure 13 shows that different current treatment duration has almost no effect on the uniformity of the interference distribution, which, in turn, further confirms that the temperature level in the connection area is the main factor determining the interference.

### 3.3. CFRP Damage

**Figure 14:**
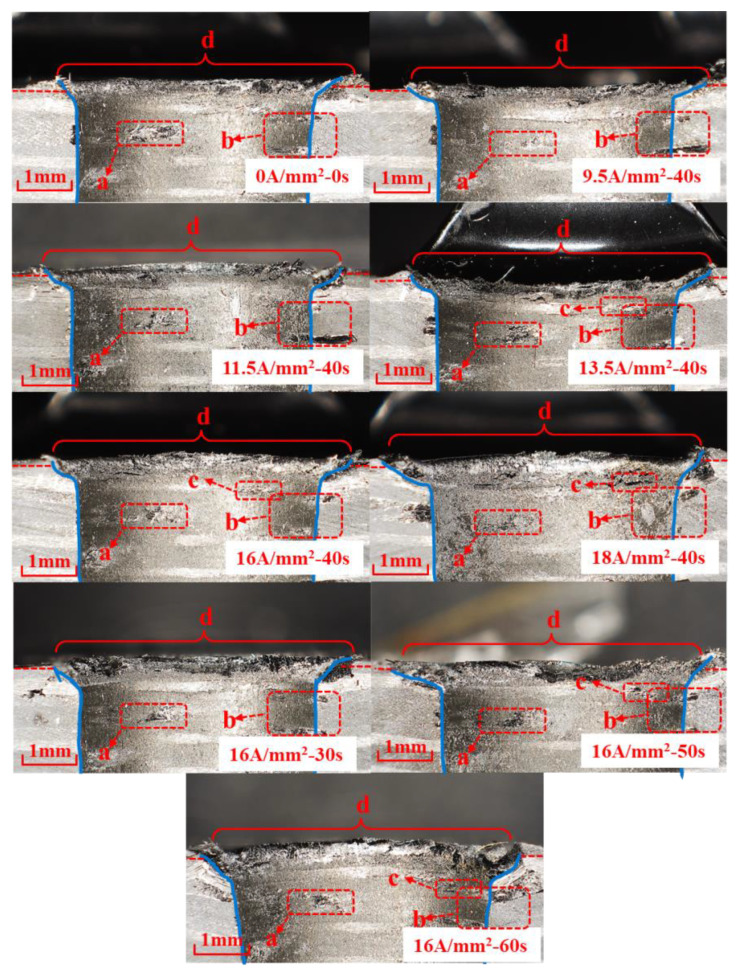
Macro appearance of CFRP connection area under different electrical parameters. a–d represents different types of hole wall damage.

**Figure 15:**
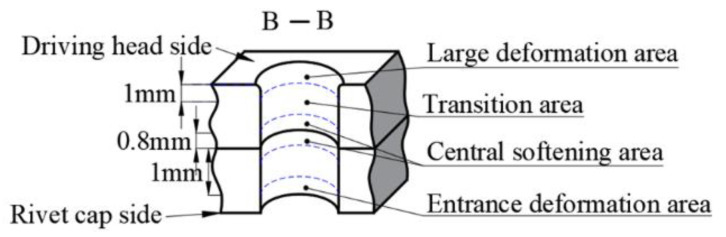
Schematic diagram of area division in the hole.

The riveted joint was cut open to observe the damage around the CFRP hole, near the driving head side. The macro appearance of the CFRP connection area is shown in Figure 14. Corresponding to the results of the interference analysis, the CFRP hole wall has a trumpet shape with a large opening near the driving head side. Furthermore, there are different degrees of CFRP material extrusion on the driving head side, resulting in radial crushing at different heights on the outlet side, as shown in the “d” region of Figure 14; this situation also occurs in conventional riveting [26]. As a benchmark, the size of the driving head and the burr height of the conventional riveting joint meet the relevant aviation standards [27], and the damage degree is regarded as within the normal range. In order to analyze the type of damage in each area, the area inside the hole is divided, as shown in Figure 15. The damage at “a” and “b” is caused during the drilling and sample cutting process.

**Figure 16:**
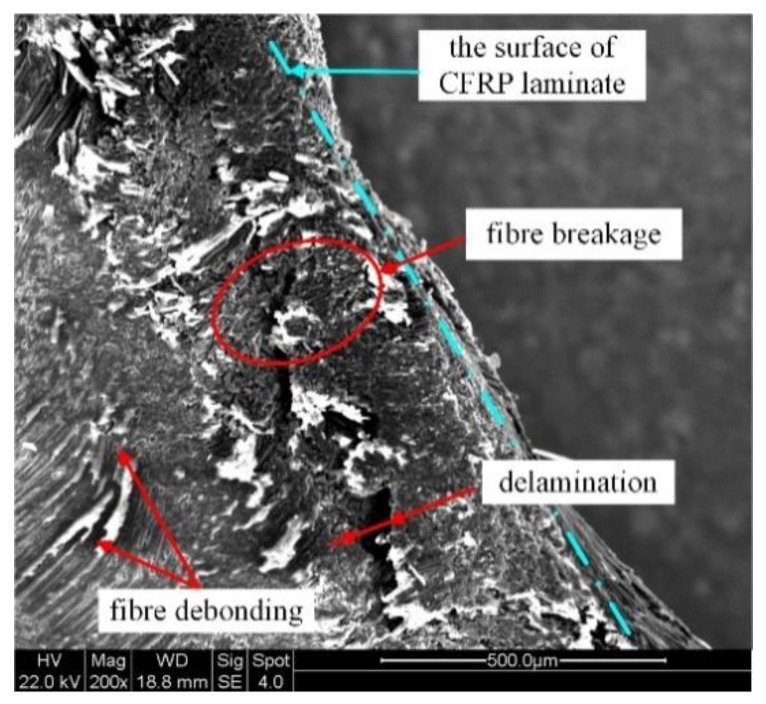
Damage appearance at position “d”.

It can be seen that as current density and action time increase, the level of interference increased, resulting in a continuous increase in the material extrusion area at the exit of the driving head side. It was most serious under electrical parameters of 18 A/mm^2^-40 s, 16 A/mm^2^-30 s, 16 A/mm^2^-50 s and 16 A/mm^2^-60 s. The appearance of damage in region “d” is shown in Figure 16. Under the combined action of high temperature and large amount of interference, the damage in region “d” produces a variety of damage forms. Under the softening action of high temperature, the strength of branched chains in the polymer decreases, the breakage rate of molecular bonds accelerates, and the bonding strength between the matrix and fiber weakens [28]. The extrusion force generated by large interference compacts the fibers near the transition zone, while the relative dislocation motion between the direction of motion in the metal filling hole and the direction of extrusion of CFRP material leads to the debonding of the carbon fibers. The carbon fiber’s thermal environment of a large deformation area near the outlet is different, because the temperature is lower and the relative movement of material extrusion is stronger, the bending behavior of fiber is more obvious, and the inter laminar tearing stress in the corresponding lamination increases, and the failure forms are mainly fiber breakage and delamination.

**Figure 17:**
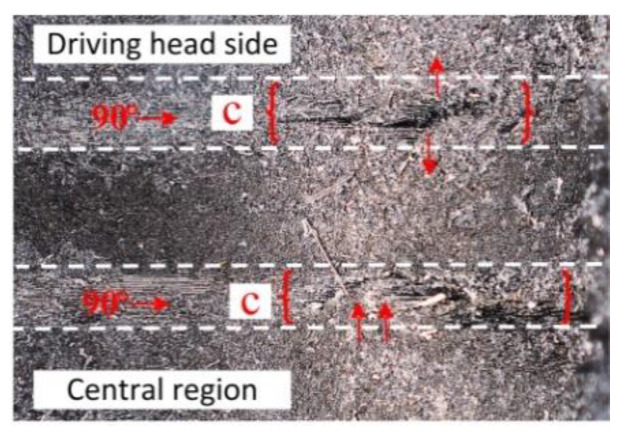
Damage appearance at position “c”.

In the joint riveted a with high current density, the damage position at “c” is all in the 90° layer. The damage position at “c” in a single CFRP plate is shown in Figure 17, and there are two examples of damage in the 90° layer. Thickness separation occurred in the c_1_ region, and thickness healing occurred in the c_2_ region, which was caused by the high temperature environment and the increase in the interference amount in the current assisted process, which showed softening of the resin matrix and weakening of interlayer strength. The interference values at different hole depths are different. For example, c_2_ near the center has a small amount of interference. High temperature leads to serious softening, enhanced resin fluidity, and the matrix of 45° layer fiber transfers to the 90° layer under radial pressure. However, c_1_ is close to the outlet, with a large amount of interference and obvious mechanical interference behavior, which exceeds the thermal softening ability of the resin matrix to compromise the compression deformation and presents obvious thick-directional separation.

**Figure 18:**
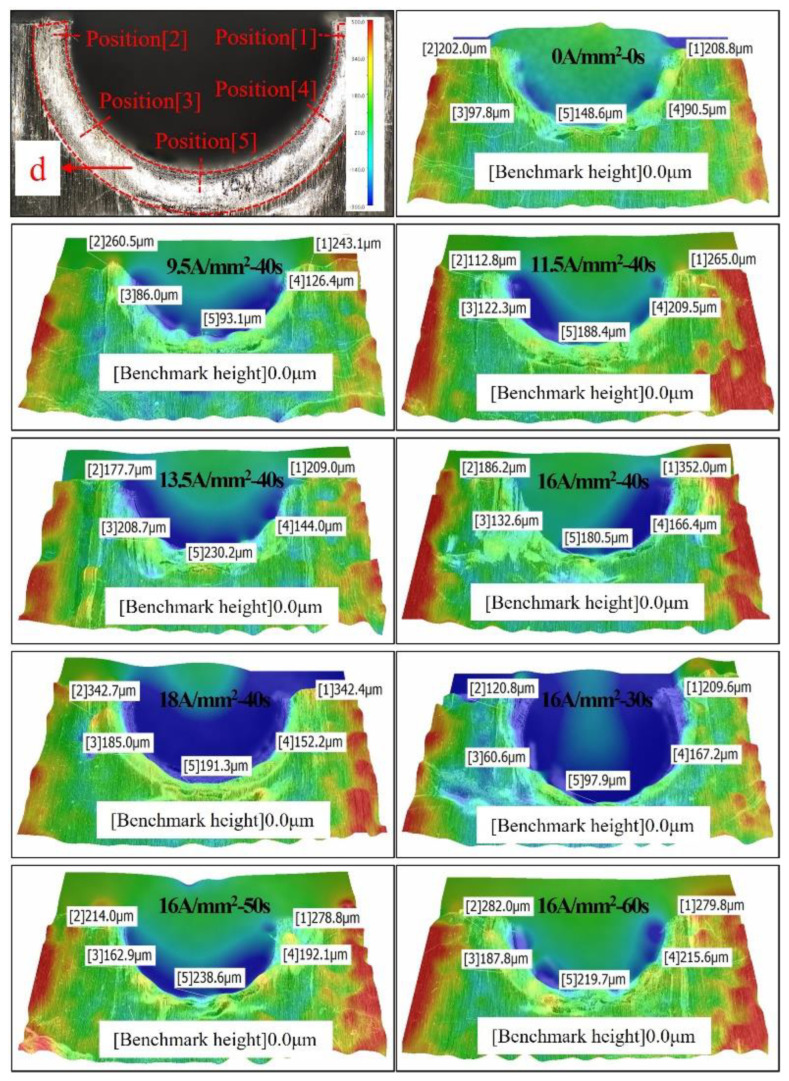
The height cloud diagram of outlet damage.

In order to quantify and analyze the height of burr at the exit of the CFRP hole, the exit area on the side of the driving head is scanned with an ultra-depth three-dimensional microscope. The height cloud diagram of outlet damage is shown in Figure 18. The zero point of reference height was selected as the flat area of the CFRP plate, and the material stacking height at five points on the semicircle of the hole was measured. The height data are shown in Table 7. With the increase in current density and duration, the temperature at the outlet increases, the damage range and degree also increase. The average height of damage caused by radial compression around the hole wall also gradually increases. However, the damage height of all groups does not exceed 0.4 mm, specified in the standard HB/Z223.21-2003. Under the riveting parameters of 9.5 A/mm^2^-40 s and 16 A/mm^2^-30 s, the damage height on the driving head side is basically the same as that of ordinary riveting. When the current density exceeds 16 A/mm^2^ or the action time exceeds 30 s, the damage height caused by radial compression will increase sharply.

## 4. Conclusions

The new technology of current-assisted riveting was used to assemble CFRP laminate, and the force, displacement and temperature rise behavior during the process were monitored. The interference law in the connection area and the CFRP damage mechanism were further analyzed quantitatively. The results are summarized as follows:

(1) Current-assisted technology can significantly improve the plasticity of a rivet, and the yield strength can be reduced by 10% to 20%, when the formed pier head is qualified. With the increase in current density, the softening effect of the rivets increases significantly, and there is a threshold for the effect of electrical duration on improvement of plasticity.
(2) The temperature of the riveting process under the electric heating environment rises rapidly and shows a saturation value. The maximum saturation temperature directly affects the softening degree of CFRP in the connection area and affects the overall interference distribution. The non-uniform distribution of temperature in the CFRP hole affects the uniformity of the interference fit formation. When the current density is 9.5 A/mm^2^-40 s, the uniformity of the interference is increased by about 30%.
(3) The most easily damaged position of the new riveting technology is the exit of the driving head, which is the same as that of conventional riveting. Large deformation results in a certain degree of crushing of the radial hole wall, but the material accumulation height formed by extrusion meets the standard requirements. Under the action of high current density and long duration, the damage in the CFRP hole is aggravated, and the damage at different depths of hole will show the damage form of different dominant mechanisms due to the level of temperature and interference.

## Figures and Tables

**Table 1:** Experimental object and related parameters.

Objects	Materials	Types	Dimensions(mm)
Rivets	Ti45Nb	button head	Φ4 × 10.4
CFRP plates	T700-50% Epoxy resin-50%	[0°/45°/−45°/90°] _S_	2.5/2.5

**Table 2:** Experimental program.

Factors	Levels					
	1	2	3	4	5	6
Current density (A) A/mm^2^	0	9.5	11.5	13.5	16	18
Action time (B) s	0	30	40	50	60	-

**Table 3:** Yield stress at different current densities.

Experiment Number	Current Density (A/mm^2^)	Yield Stress σ_s_ (MPa)	
		Average	Standard Deviation
A_1_B_3_	0	640	10.8
A_2_B_3_	9.5	600	8.6
A_3_B_3_	11.5	568	3.7
A_4_B_3_	13.5	526	6.4
A_5_B_3_	16	517	2.4
A_6_B_3_	18	493	6.9

**Table 4:** Yield stress at different electric action times.

Experiment Number	Electrical Action Time (S)	Yield Stress σ_s_ (MPa)	
		Average	Standard Deviation
A_5_B_1_	0	640	10.80123
A_5_B_2_	30	554	5.88784
A_5_B_3_	40	517	4.32049
A_5_B_4_	50	515	8.64098
A_5_B_5_	60	511	5.09901

**Table 5:** Interference fit level distribution (mm).

Experiment Number	Position P_1_	Position P_2_	Position P_3_	Position P_4_	Position P_5_	Mean $\;\overset{¯}{\Delta }$
A_1_B_3_/A_5_B_1_	0.095	0.055	0.025	0.035	0.045	0.051
A_2_B_3_	0.100	0.05	0.035	0.055	0.055	0.06
A_3_B_3_	0.135	0.065	0.035	0.055	0.060	0.0725
A_4_B_3_	0.155	0.065	0.035	0.060	0.065	0.07875
A_5_B_3_	0.175	0.080	0.055	0.070	0.075	0.095
A_6_B_3_	0.205	0.105	0.065	0.085	0.095	0.115
A_5_B_2_	0.140	0.055	0.040	0.055	0.055	0.069
A_5_B_4_	0.270	0.135	0.085	0.085	0.090	0.133
A_5_B_5_	0.270	0.155	0.095	0.105	0.100	0.145

**Table 6:** Coefficients required for model solution.

Symbol	Unit	Value
*d*	mm	4
*K*_f_	W/m·K	0.51
*ρ*_*s*_	Ω·m^2^/m	1.7 × 10^−6^
$\;\overset{¯}{\varepsilon }$	-	0.4
*f*	Hz	33,000
*D*	s	3.03 × 10^−4^
*J*	A/mm^2^	9.5

**Table 7:** Height of crush damage on driving head side (μm).

Experiment Number	Position [1]	Position [2]	Position [3]	Position [4]	Position [5]	Mean $\;\overset{¯}{h}$
A_1_B_3_/A_5_B_1_	208.8	202.0	97.8	90.5	148.6	149.54
A_2_B_3_	243.1	260.5	86.0	126.4	93.1	161.82
A_3_B_3_	265.0	112.8	122.3	209.5	188.4	179.6
A_4_B_3_	209.0	177.7	208.7	144.0	230.2	193.92
A_5_B_3_	352.0	186.2	132.6	166.4	180.5	203.54
A_6_B_3_	342.4	342.7	185.0	152.2	191.3	242.72
A_5_B_2_	209.6	120.8	60.6	167.2	97.9	131.22
A_5_B_4_	278.8	214.0	162.9	192.1	238.6	217.28
A_5_B_5_	279.8	282.0	187.8	215.6	219.7	236.98

## Data Availability

The data presented in this study are available from the corresponding authors upon reasonable request.
